# Development of a molecular platform for HTLV confirmatory diagnosis: importance of the internal amplification control

**DOI:** 10.1186/1742-4690-8-S1-A251

**Published:** 2011-06-06

**Authors:** Maurício C Rocha-Junior, Rodrigo Haddad, Evandra S Rodrigues, Osvaldo M Takayanagui, Dimas T Covas, Simone Kashima

**Affiliations:** 1 Regional Blood Center of Ribeirão Preto, Ribeirão Preto, Brazil; 2 Faculty of Pharmaceutical Sciences of Ribeirão Preto, University of São Paulo, Ribeirão Preto, Brazil; 3 Faculty of Medicine of Ribeirão Preto, University of São Paulo, Ribeirão Preto, Brazil

## Background

Brazil may harbor the largest absolute number of HTLV-1-infected individuals worldwide. The current HTLV diagnosis is based mainly on antibodies detection. However, these tests exhibit high proportion of indeterminate results. Several real-time PCR techniques have been developed for the detection of HTLV-1/2. However, up to day the major drawbacks of HTLV-1/2 molecular diagnosis are the lack of standard molecular tests and the absence of suitable internal amplification control (IAC). The aim of this study was to develop a multiplex qualitative real-time PCR for the simultaneous detection and discrimination of HTLV-1/2 and to design an IAC for reaction monitoring.

## Methods

After multiple sequence alignments of the full genomes of HTLV-1/2 subtypes, a conserved tax region was chosen for the design of specific primers and probes. The IAC was generated after the annealing of synthetic nucleotide sequences and cloned into TOPO TA® vector. MT-2 and Gu cell lines were used as positive controls for HTLV-1 and HTLV-2, respectively.

## Results

The developed multiplex real-time reaction detected both HTLV-1 and HTLV-2 (105 to 101 copies/reaction) at the presence of IAC in the same reaction. Analytical sensitivity was 1.2 copies/reaction for HTLV-1 and 19.1 copies/reaction for HTLV-2. The analytical sensitivity analysis was performed in singleplex format.

## Conclusion

The detection of HTLV-1/2 and IAC by multiplex real-time PCR was efficient. The developed IAC is suitable for the molecular diagnosis and its presence ensures that the negative results are not due to failure in pre-PCR procedures.

